# Chlojaponilactone B Attenuates Lipopolysaccharide-Induced Inflammatory Responses by Suppressing TLR4-Mediated ROS Generation and NF-κB Signaling Pathway

**DOI:** 10.3390/molecules24203731

**Published:** 2019-10-16

**Authors:** Shaoxia Ye, Qiyao Zheng, Yang Zhou, Bai Bai, Depo Yang, Zhimin Zhao

**Affiliations:** 1 School of Pharmaceutical Sciences, Sun Yat-sen University, Guangzhou 510006, Guangdong, China; yeshx9@mail2.sysu.edu.cn (S.Y.); zhouy596@mail2.sysu.edu.cn (Y.Z.); baib6@mail2.sysu.edu.cn (B.B.);; 2 Guangdong Technology Research Center for Advanced Chinese Medicine, Guangzhou 510006, Guangdong, China

**Keywords:** chlojaponilactone B, *Chloranthus japonicus*, inflammation, TLR4, NF-κB, ROS, RNA-seq

## Abstract

The lindenane-type sesquiterpenoid chlojaponilactone B (**1**), isolated from *Chloranthus japonicus*, has been reported to possess anti-inflammatory properties. The present study aimed to further explore the molecular mechanisms underlying the anti-inflammatory activity of **1**. RNA-seq analyses revealed the significant changes in the expression levels of genes related to multiple inflammatory pathways upon treatment of lipopolysaccharide (LPS)-induced RAW 264.7 murine macrophages with **1**. Real time PCR (RT-PCR) and Western blotting were used to confirm the modulations in the expression of essential molecules related to inflammatory responses. Compound **1** inhibited toll like receptor 4 (TLR4) and myeloid differentiation factor 88 (MyD88) activation upon LPS stimulation, influencing the expression of NF-κB and pro-inflammatory mediators. Molecular docking studies showed that **1** bound to TLR4 in a manner similar to that of TAK-242, a TLR4 inhibitor. Moreover, our results showed that **1** suppressed inflammatory responses by inhibiting TLR4 and subsequently decreasing reactive oxygen species (ROS) generation, downregulating the NF-κB, thus reducing the expression of the pro-inflammatory cytokines iNOS, NO, COX-2, IL-6 and TNF-α; these effects were similar to those of TAK-242. We proposed that **1** should be considered as a potential anti-inflammatory compound in future research.

## 1. Introduction

Inflammation is a first defense initiated by the immune system to noxious stimuli, infections and tissue injury [1]. Appropriate inflammatory responses are protective for the body, but excessive responses lead to inflammation related diseases such as cancer, cardiovascular diseases and rheumatoid arthritis [2,3]. Although inflammation has been investigated intensively in recent years, owing to its biological complexity and its close relationship with diverse pathological conditions, it is still a therapeutic challenge to develop novel anti-inflammatory drugs with high efficacy and low side effects [4,5].

TLRs are key transmembrane receptors that play a significant role in recognizing microbial infections in mammals [6]. TLR4, a key member of the TLR family, participates in the intrinsic immune response by recognizing pathogen-associated molecular patterns (PAMPs) [7]. After recognizing lipopolysaccharide (LPS), and recruiting its adaptor protein MyD88, TLR4 triggers the activation of the transcription factor NF-κB, resulting in an increase in the expression of pro-inflammatory cytokines iNOS, NO, COX-2, IL-6 and TNF-α [8,9,10]. The dysregulation of TLR4 signaling contributes to a series of acute and chronic human diseases such as septic shock [11], rheumatoid arthritis [12], atherosclerosis [13] and cancer [14]. Thus, TLR4 has become an important therapeutic target, and blocking the TLR4-mediated signaling pathways may be a more effective approach to treating inflammation.

Chlojaponilactone B (**1**) is a lindenane-type sesquiterpenoid and has been isolated from the perennial herbaceous plant *Chloranthus japonicus*, which has a long history in the traditional Chinese medicine (TCM) for treating fractures, neurasthenia, pulmonary tuberculosis, rheumatic arthralgia and traumatic injuries [15]. Moreover, *C. japonicus* was used for the treatment of boils, dermatological issues and enteric fever in South Korea [16]. In our previous study, we reported that **1** has anti-inflammatory properties [1]. However, the detailed mechanism of the anti-inflammatory effect of **1** remains unclear. Therefore, we herein used RNA sequencing (RNA-seq) to clarify the molecular mechanisms underlying the anti-inflammatory activity of **1**. RNA-seq analyses revealed significant changes in the expression levels of genes associated with inflammatory pathways upon treating LPS-induced RAW 264.7 murine macrophages with **1**. Molecular docking studies showed that **1** bound to TLR4 in a manner similar to that of TAK-242, a well-known TLR4 inhibitor. Our in vitro study revealed that **1** modulated the inflammatory response by inhibiting TLR4, leading to ROS generation inhibition and NF-κB signaling pathway suppression, ultimately decreasing the expression of the pro-inflammatory cytokines iNOS, NO, COX-2, IL-6 and TNF-α. Herein, we reported TLR4-mediated anti-inflammatory effects of **1**.

## 2. Results

### 2.1. Distinct Transcriptome Profile upon Treating LPS-induced RAW 264.7 Macrophages with ***1***, as Determined by RNA-seq

**Figure 1:**
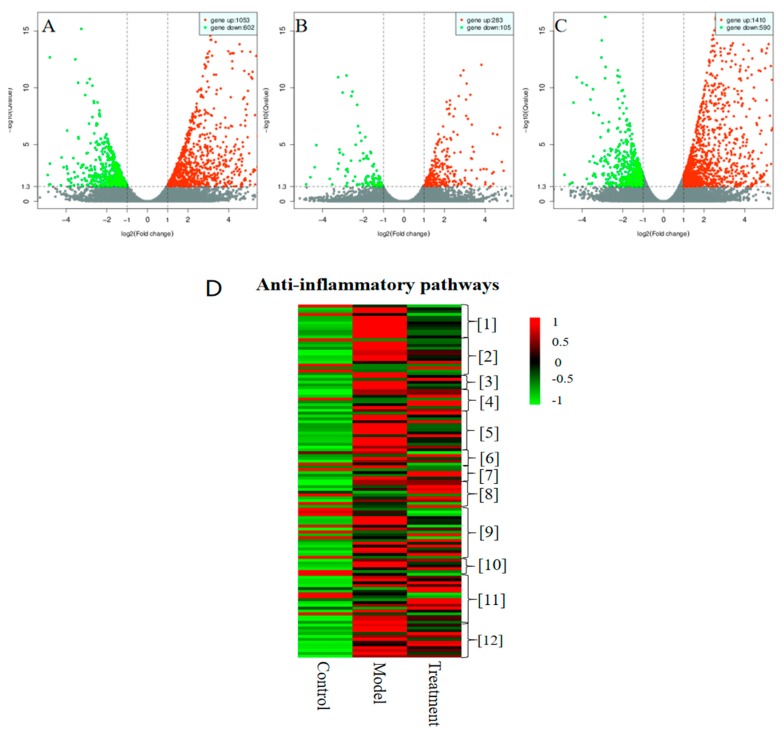
Distinct transcriptome profile obtained upon treating RAW 264.7 macrophages with chlojaponilactone B (**1**), as determined by RNA-seq. Horizontal coordinates represent variations in mRNA expression levels, and longitudinal coordinates represent significant changes in mRNA expression levels. Red and green dots in the Volcano plot indicate mRNAs with increased and decreased expression levels, respectively. Red and green squares in the Heat map indicate mRNAs with increased and decreased expression levels, respectively (see color scale). RAW 264.7 macrophages treated with LPS (1 μg/mL) for 24 h served as the model group and those treated with **1** (3.5 μM) and LPS (1 μg/mL) served for 24 h as the treatment group. Cells cultured with 0.05% DMSO for 24 h served as the control group. (**A**) Control group vs. model group. (**B**) Model group vs. treatment group. (**C**) Control group vs. treatment group. (**D**) Heat map analysis of anti-inflammatory pathway profiles for the three groups. Three independent experiments were performed for each group, followed by data analyses.

RNA-seq analysis was performed using LPS-induced RAW 264.7 murine macrophages treated with **1** at concentration of 3.5 μM. Differentially expressed transcripts were identified based on the criteria of fold change >1.5, and *p* < 0.05. A total of 4954 differentially expressed mRNAs were found in the model group (LPS-treated cells) relative to the control group (DMSO vehicle-treated cells): the expression levels of 2937 mRNAs increased, while that of 2017 mRNAs decreased. A total of 1229 differentially expressed mRNAs were found in the treatment group (LPS plus **1**-treated cells) relative to the model group: the expression levels of 895 mRNAs increased, while that of 334 mRNAs decreased. A total of 5164 differentially expressed mRNAs were found in the treatment group relative to the control group: the expression levels of 3594 mRNAs increased, while that of 1570 mRNAs decreased. Volcano plots demonstrating these results are shown in Figure 1A–C; RNA-seq profiles for the three groups are displayed as a heat map in Figure 1D. Genes related to 12 inflammatory pathways were significantly differentially expressed, as elucidated via Kyoto Encyclopedia of Genes and Genomes (KEGG) analysis (Table 1). RNA-seq data showed significant differences in the expression levels of genes encoding inflammatory receptors (TLR4 and MyD88) and inflammatory markers.

### 2.2. Effects of ***1*** on the mRNA Expression of TLR4, MyD88 and Pro-Inflammatory Mediators in LPS- Induced RAW 264.7 Macrophages

**Figure 2:**
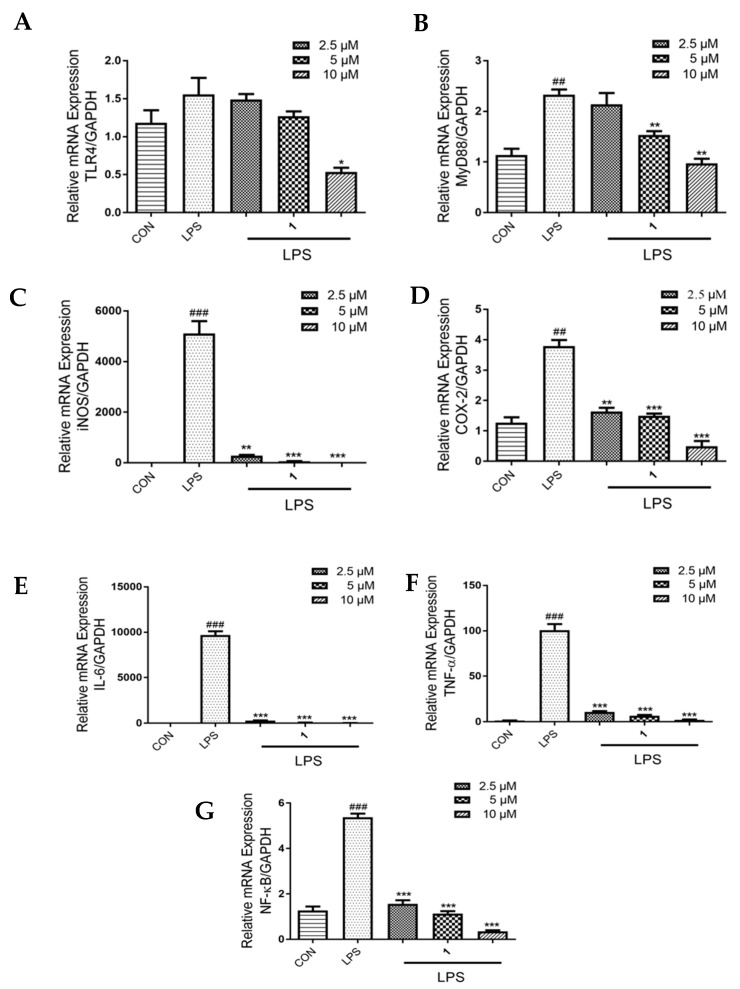
Effects of **1** on the expression of (**A**) TLR4, (**B**) MyD88, (**C**) iNOS, (**D**) COX-2, (**E**) IL-6, (**F**) TNF-α and (**G**) NF-κB. RAW 264.7 cells were incubated with the indicated concentrations of **1** (2.5, 5 or 10 μM) and LPS (1 μg/mL) for 24 h, as determined by RT-PCR assay. GAPDH was used as the internal control. Values are representative of three independent experiments. ^##^ *p* < 0.01, ^###^ *p* < 0.005 vs. control cells. * *p* < 0.05, ** *p* < 0.01, *** *p* < 0.005 vs. LPS-induced cells.

To explore the expression of inflammatory receptors and pro-inflammatory mediators upon treating LPS-induced macrophages with **1**, real time PCR (RT-PCR) was performed. The obtained results showed that exposure to LPS upregulated the mRNA expression of TLR4 and MyD88, but treatment with **1** attenuated this upregulation in a dose-dependent manner. Furthermore, we noted that the increased expression of iNOS, COX-2, IL-6, TNF-α and NF-κB in response to LPS exposure was markedly decreased (*p* < 0.01) upon treatment with **1** in a dose-dependent manner in LPS-induced RAW 264.7 macrophages (Figure 2).

### 2.3. Effects of ***1*** on the Protein Expression of TLR4 and MyD88 in LPS-Induced RAW 264.7 Macrophages

**Figure 3:**
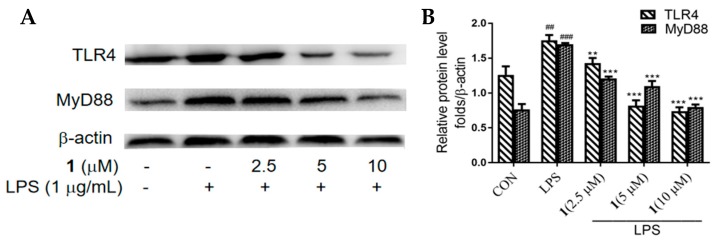
Effect of **1** on TLR4 and MyD88 expression in LPS-induced RAW 264.7 macrophages, as determined by Western blotting. Cells were incubated with various concentrations of **1** (2.5, 5 or 10 μM) and LPS (1 μg/mL) for 24 h. β-actin was used as the internal control. (**A**) Results are presented as representative of three independent experiments and summarized in the bar graphs. (**B**) Data are presented as means ± SD of three independent experiments. ^##^ *p* < 0.01, ^###^ *p* < 0.005 vs. control cells. ** *p* < 0.01, *** *p* < 0.005 vs. LPS-induced cells.

To further explore whether **1** influenced inflammatory receptors at the protein level, we performed Western blotting to determine the levels of TLR4 and MyD88 in LPS-induced RAW 264.7 macrophages after treating with **1**. The enhanced levels of TLR4 and MyD88 on the stimulation of LPS were decreased by treatment of **1** in a dose-dependent manner (*p* < 0.01) (Figure 3).

### 2.4. Affected TLR4 Signaling by Inhibiting TLR4 Protein

**Figure 4:**
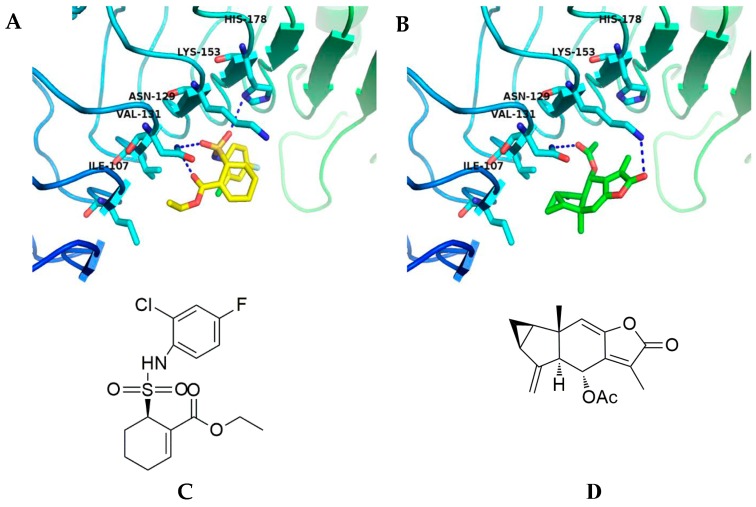
In silico studies to determine binding patterns of TAK-242 or **1** with TLR4. (**A**) TAK-242 (yellow) formed three hydrogen bonds with Asn129 and His178 in the binding site. (**B**) **1** (green) formed two hydrogen bonds with Asn129 and Lys153 in the binding site. Hydrogen bonds are depicted via dashed blue lines. Chemical structures of (**C**) TAK-242 and (**D**) **1**. Docking poses of TAK-242 or **1** were simulated using the crystal structure of the mouse TLR4 protein (PDB: 2Z64).

TAK-242 is a specific, small-molecule inhibitor of the intracellular signaling pathways of TLR4, and it limits NF-κB-mediated pro-inflammatory cytokines generation [17]. To explore the inhibitory mechanism of **1** or TAK-242 against TLR4, we performed molecular docking studies to predict the binding patterns of **1** or TAK-242 with TLR4. The calculated binding affinity of **1** (-CDOCKER_INTERACTION_ENERGY = 22.3062) was approximate to that of TAK-242 (-CDOCKER_INTERACTION_ENERGY = 24.7947). As shown in Figure 4, TAK-242 formed three hydrogen bonds with Asn129 and His178, while **1** formed two hydrogen bonds with Asn129 and Lys153 in the *β*-sheet in the binding sites of the TLR4 protein. Results of molecular docking studies clearly indicated that **1** interacts with TLR4 via hydrogen bonds formed with the acetyl and carbonyl groups, while TAK-242 formed hydrogen bonds with TLR4 via the acyl and sulfonic groups.

### 2.5. Effects of ***1*** on NO Production and ROS Generation in LPS-Induced RAW 264.7 Macrophages

**Figure 5:**
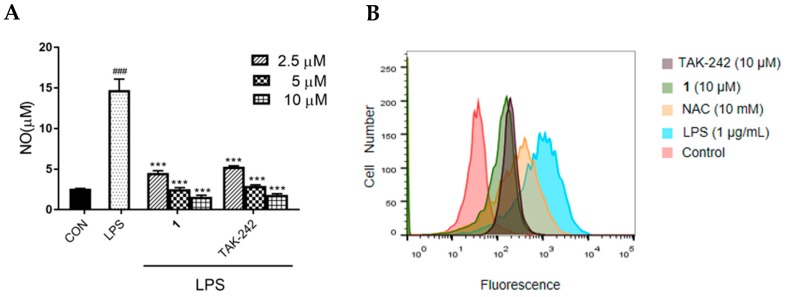
Effect of **1** on NO and ROS production in LPS-induced RAW 264.7 macrophages. (**A**) NO and (**B**) ROS was measured using the Griess systems and flow cytometry, respectively. For NO measurement, cells were incubated with varied concentrations of **1** (2.5, 5 or 10 μM) or TAK-242 (2.5, 5 or 10 μM) and treated with LPS (1 μg/mL) for 24 h. For ROS determination, cells were incubated with **1** (10 μM), TAK-242 (10 μM) or NAC (10 mM) and treated with LPS (1 μg/mL) for 24 h. Results are representative of three independent experiments. ^###^ *p* < 0.005 vs. control cells, *** *p* < 0.005 vs. LPS-induced cells.

For investigating the TLR4-mediated anti-inflammatory effects of **1**, we first examined the inhibitory effect of **1** on NO production in LPS-induced RAW264.7 macrophages. As shown in Figure 5A, NO production was observed after exposure to LPS, whereas treatment with **1** caused a sustained decrease in LPS-induced NO production in a dose-dependent manner (*p* < 0.005). Meanwhile, TAK-242 exerted significant decrease in LPS-induced NO production in a dose-dependent manner (*p* < 0.005). The effect of **1** in decreasing NO production was similar to that of TAK-242. The tested concentration of **1** and TAK-242, determined using MTT assay, showed no toxicity (Figure S1, Supplementary Materials). As shown in Figure 5B, ROS generation were markedly increased upon exposure to LPS, and significantly decreased after treatment with **1** or TAK-242. The effect of **1** in inhibiting ROS generation was better than that of TAK-242 and the positive control, N-acetylcysteine (NAC).

### 2.6. Effects of ***1*** on NF-κB Phosphorylation, and the Expression of iNOS, COX-2, IL-6 and TNF-α in LPS-Induced RAW 264.7 Macrophages

**Figure 6:**
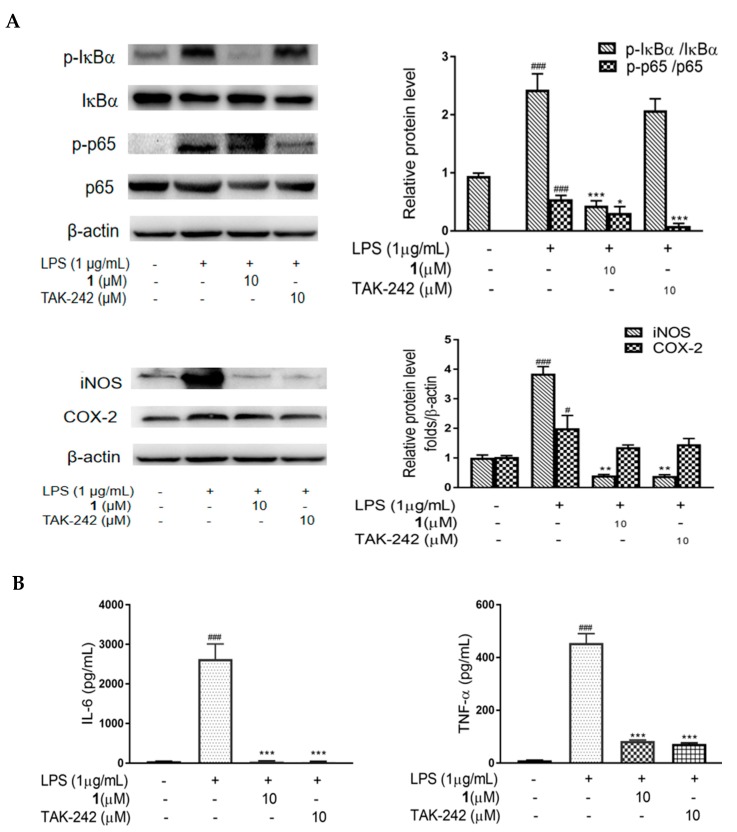

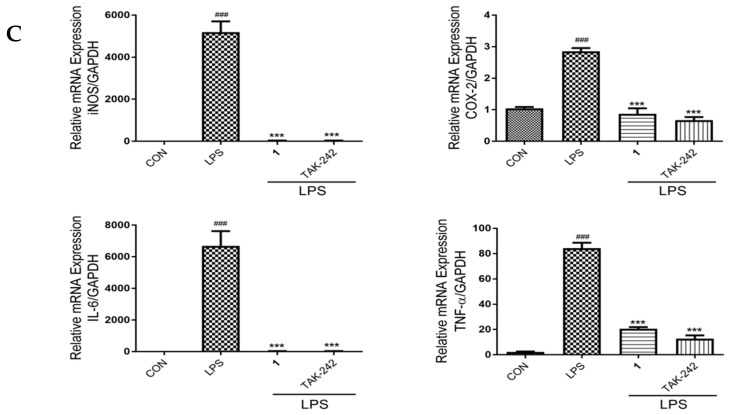
Effect of **1** on phosphorylation of NF-κB subunits, and the expression of pro-inflammatory cytokines in LPS-induced RAW 264.7 macrophages. Cells were incubated with **1** (10 μM) or TAK-242 (10 μM) and LPS (1 μg/mL) for 24 h. (**A**) Western blotting was performed to detect the expression of IκBα, p-IκBα, p65, p-p65, iNOS and COX-2. β-Actin served as the internal control. Quantification of immunoreactive protein bands is shown via bar graphs. (**B**) ELISA was determined to measure the production of IL-6 and TNF-α. (**C**) RT-PCR was performed to detect the mRNA levels of iNOS, COX-2, IL-6 and TNF-α. GAPDH was used as the internal control. Values are representative of three independent experiments. ^#^ *p* < 0.05, ^###^ *p* < 0.005 vs. control cells. * *p* < 0.05, ** *p* < 0.01, *** *p* < 0.005 vs. LPS-induced cells.

To further investigate if **1** is involved in TLR4-mediated NF-κB signaling pathway activation, the expression of NF-κB was evaluated. As shown in Figure 6A, LPS stimulation resulted in a notable increase (*p* < 0.005) in p65 and IκBα phosphorylation, while treatment with **1** (10 μM) exerted an inhibitory effect on the expression of p65 (*p* < 0.05) and IκBα (*p* < 0.005); TAK-242 (10 μM) exerted an inhibitory effect on the expression of p65 (*p* < 0.005), while exerting an slight inhibitory effect on the expression of IκBα. The effect of **1** in degrading the phosphorylation of p65 was inferior to that of TAK-242, but the effect of **1** in degrading the phosphorylation of IκBα was better than that of TAK-242. In addition, to evaluate the effect of **1** and TAK-242 in inhibiting the expression of inflammatory cytokines in LPS-induced RAW264.7 cells, we performed Western blotting, ELISA and RT-PCR. Compound **1** at 10 μM significantly (*p* < 0.01) attenuated the LPS-induced increase in the expression of iNOS, IL-6 and TNF-α, while slightly down-regulated the level of COX-2. The effect was similar with that of 10 μM TAK-242 (Figure 6A–C).

## 3. Discussion

Inflammation is a host response of the body to remove harmful stimuli. Acute inflammation is a reaction during infectious challenge and tissue injury, whereas chronic inflammation is a persistent reaction caused by tissue damage [18]. LPS is a complex glycolipid in the outer membrane of Gram-negative bacteria, which are utilized to induce inflammatory [19]. Although **1** is known as regulating inflammatory responses via NF-κB activation in LPS-stimulated macrophages and LPS-treated mice, the detailed mechanism involving NF-κB pathway remains unclear. To this end, we employed RNA-seq technology to explore the molecular mechanism of **1**. RNA-seq is usually used for prenatal screening, genetic disease diagnosis, tumor mutant gene detection and targeted-therapy [20,21,22,23]. The rapid development of RNA-seq has provided a novel method to investigate the mechanisms underlying TCM, facilitating the interpretation of the effects of TCM at a molecular level. In this study, RNA-seq analysis revealed the significant changes in the transcriptome of multiple pathways driven by **1** treatment in LPS-induced RAW 264.7 cells. Inhibition of TLR-4 and MyD88 contributed to the anti-inflammatory effect of **1**, in addition to inhibition of inflammatory markers iNOS, COX-2, IL-6, TNF-α and NF-κB. These results were consistent with those of our previous study, wherein we observed that **1** decreased the expression of inflammatory markers.

As an important category of pattern recognition receptors, TLRs could recognize LPS and transduce a series of signals through NF-κB signaling pathway to upregulate pro-inflammatory cytokine expression [8]. TLR4 is also required for the activation of pro-inflammatory cellular signaling pathways in response to endogenous molecules [19,24]. Accumulating evidence has implicated TLR4 as a crucial signal transducing receptor that recognizes PAMPs such as LPS thereby inducing the secretion of inflammatory cytokines and activating the innate immune system [7,8,19]. In general, TLR4 activates the downstream signaling cascades via MyD88-dependent pathways [25]. The decreased activation of TLR4/MyD88 could attenuate the binding of LPS and thereby down-regulated the expression of downstream messenger molecules and related inflammatory genes [26]. In our study, **1** dose-dependently suppressed LPS-induced expression of TLR4 and MyD88, which indicates that the anti-inflammatory effect of **1** on LPS stimulation is associated with TLR4-mediated inflammatory signaling pathway.

The structure of **1** has been determined to be (1α,3α,5α,6α)-6-(acetyloxy) lindena-4 (15),7 (11),8-trieno-12,8-lactone [27]. In our previous study, by analysis of the structure-activity relationship, we supposed that the anti-inflammatory effect of **1** depends on the double bond between C-8 and C-9 and acetyl group at C-6. The acyl group of **1** is similar to the TLR4 inhibitor, ethyl (6R)-6-[N-(2-chloro-4-fluorophenyl) sulfamoyl] cyclohex-1-ene-1-carboxylate (TAK-242). TAK-242 possesses potent anti-inflammatory properties; it has been successful in pre-clinical trials but was withdrawn from phase III clinical trials due to lack of efficacy [28,29]. Therefore, identifying novel TLR4 signaling inhibitors that can be used for therapeutic purposes is urgently needed. In silico analysis of **1** showed that it potentially binds to the TLR4 binding site in a manner similar to that of TAK-242. In this study, the results of molecular docking studies clearly indicated that **1** interacts with TLR4 via hydrogen bonds formed with the acetyl and carbonyl groups, rather than via the double bond between C-8 and C-9. We considered that the acyl group of **1** or TAK-242 serves as an important pharmacophore for the anti-inflammatory effect, and **1** may function as a novel TLR4 signaling inhibitor.

It was commonly recognized that TLR4 exerted anti-inflammatory effects by the phosphorylation of transcriptional regulators, particularly NF-κB [8]. Upon certain stimuli, such as LPS, NF-κB was phosphorylated and transported into the nucleus to regulate a variety of inflammatory cytokines expression, including iNOS, NO, COX-2, IL-6 and TNF-α [30]. NF-κB pathway has long been considered as a prototypical pro-inflammatory signaling pathway, largely based on its role in upregulating the expression of pro-inflammatory genes [31]. The most common NF-κB dimer is the p65/p50 heterodimer, which is bound to an inhibitor of NF-κB (IκBα) and is inactive in the cytoplasm [1]. After LPS stimulation, the phosphorylation of p65 and IκBα enhances NF-κB activation during inflammatory processes [1,32]. TLR4 activates the downstream NF-κB signaling pathway and upregulates pro-inflammatory cytokines expression after recognizing LPS [33]. Pro-inflammatory cytokines play a fundamental role during the propagation of several inflammatory ailments, including ulcerative colitis and acute lung injury [34], and have been reported to be upregulated during the inflammation process; therefore, compounds that down-regulate their expression are considered to possess anti-inflammatory properties. Meanwhile, TLR4 stimulation with LPS reportedly induces ROS generation [35]. ROS are associated with the development of many pathological conditions because of oxidative damage, which triggers NF-κB-p65 activation by increasing the phosphorylation of IκBα [36]. TLR4 can induce ROS generation, and then activate the NF-κB signaling pathway, consequently leading to the expression of pro-inflammatory cytokines [35,36]. To further investigate the molecular mechanism of **1**, we herein focused on TLR4 and its downstream signaling pathways. After the administration of **1**, the production of ROS was decreased, the phosphorylation of p65 and IκBα was inhibited, and the expression of pro-inflammatory cytokines iNOS, NO, COX-2, IL-6 and TNF-α was decreased, these effects were similar to that of TAK-242. Particularly, **1** dramatically decreased ROS generation and significantly inhibited IκBα phosphorylation. We conjectured that a decrease in ROS generation upon treating LPS-induced RAW264.7 macrophages with **1** may contribute to the inhibition of NF-κB activation; these results were consistent with reports wherein a decrease of ROS causes an inhibition of NF-κB-p65 activation [35,36]. Effects of **1** and TAK-242 were consistent with those of molecular docking studies, in which the binding affinity of **1** is approximate to that of TAK-242. It was confirmed that the ant-inflammatory effect of **1** through the NF-κB signaling pathway could be mediated by TLR4. Thus, the proper modulation of TLR4 by a natural bioactive compound could be a therapeutic concept to reduce LPS-induced inflammation.

## 4. Materials and Methods

### 4.1. General Experimental Procedures

The NMR spectra were recorded in CDCl_3_ solution on a Bruker AM-400 spectrometer at 25 °C (Bruker, Co., Rheinstetten, Germany) at 400 MHz (^1^H) or 100 MHz (^13^C). C_18_ reversed-phase silica gel (12 nm, S-50 μm, YMC Co. Ltd., Kyoto, Japan), Sephadex LH-20 gel (Amersham Biosciences, Uppsala, Sweden), and MCI gel (CHP20P, 75−150 μm, Mitsubishi Chemical Industries Ltd., Tokyo, Japan) were used for column chromatography. HPLC analysis was performed using a Shimadzu LC-20 AT equipped with an SPD-M20A PDA detector (Shimadzu, Kyoto, Japan). All solvents used were of analytical grade (Guangzhou Chemical Reagents Company, Ltd., Guangzhou, China).

### 4.2. Plant Material

The whole plants of *Chloranthus japonicus* were collected in March 2018 at Xishuangbanna, Yunnan Province, China, and authenticated by one of the co-authors (Depo, Yang). A voucher specimen (accession number: CJB201806) has been deposited at the School of Pharmaceutical Sciences, Sun Yat-sen University.

### 4.3. Extraction and Isolation

Whole *Chloranthus japonicus* plants (20 kg) were air-dried, powdered, and extracted using 95% EtOH (3 × 75 L) for 1 week each at room temperature [1]. The 95% EtOH extract was evaporated by removing the solvents to obtain the crude extract (1.5 kg), which was suspended in H_2_O (5 L). The crude extract was partitioned into petroleum ether (3 × 5 L), EtOAc (3 × 5 L), and *n*-BuOH (3 × 5 L) fractions. The EtOAc extract (485.00 g) was purified by MCI gel column chromatography (CC) by gradient elution with an MeOH/H_2_O gradient (30:70 to 100:0) to obtain five fractions (I−V). Fraction III (52.06 g) was chromatographed over a C_18_ reversed-phase (RP-18) silica gel by gradient elution with petroleum ether/EtOAc (100:0 to 75:25) to obtain six fractions (IIIa−IIIf). Fraction IIIc (1.95 g) was purified on a Sephadex LH-20 CC column using isocratic elution with CH_2_Cl_2_/MeOH (0:100) to afford three fractions (Ⅳa–Ⅳc). Finally, Fraction Ⅳb (700 mg) was purified by HPLC with MeOH/ H_2_O (70:30) to yield chlojaponilactone B (**1**) (300 mg).

### 4.4. Cell Culture

The RAW 264.7 murine macrophage cell line was purchased from the Cell Bank of Shanghai Institute of Biochemistry and Cell Biology (Chinese Academy of Sciences, Shanghai, China). The cells were cultured in Dulbecco’s Modified Eagle’s Medium (DMEM, HyClone, Logan, UT, USA) supplemented with 10% Fetal Bovine Serum (FBS, Gibco, Grand Island, NY, USA) 100 U/mL penicillin (HyClone), and 100 μg/mL streptomycin (HyClone). The cells were cultured at 37 °C, 5% CO_2_, in a humidified incubator (Thermo Fisher, San Diego, CA, USA).

### 4.5. NO Production

NO production was quantified by measuring nitrite accumulation in the culture medium using the Griess reaction [1]. RAW264.7 macrophages were cultured in 96-well plates at a density of 5.5 × 10^4^ cells/well for 24 h and then treated with **1** or TAK-242 at various concentrations with or without LPS (1 μg/mL). Wells with no test compounds received only LPS served as controls; Wells with neither any test compounds nor LPS served as blank controls. After 24 h stimulation, supernatants were obtained and mixed with an equal volume of Griess reagent (Beyotime Biotechnology, Shanghai, China). NaNO_2_ was used to generate a standard curve, and nitrite production was determined by measuring optical density at 540 nm using a microplate reader.

### 4.6. ROS Measurement

Intracellular ROS levels were measured with a 2,7-dichloro-dihydro-fluorescein diacetate (DCFH-DA) assay [37]. Cells treated with LPS (1 μg/mL) served as the model group, those treated with 0.05% DMSO served as the negative control, and those treated with **1** (10 μM), TAK-242 (10 μM) or NAC (10 mM) and LPS (1 μg/mL) served as the treatment group (all for 24 h). The cells were then treated with 10 μM DCFH-DA, followed by incubation for 30 min. The fluorescence of intracellular ROS was observed using flow cytometry.

### 4.7. RNA-seq Analysis

RAW 264.7 macrophages were cultured in six-well plates at a density of 1.0 × 10^6^ cells/plate for 24 h. The cells treated with LPS (1 μg/mL) served as the model group, those treated with **1** (3.5 μM) and LPS (1 μg/mL) served as the treatment group, and those treated with 0.05% DMSO served as the control group (all for 24 h). Cells in aforesaid three groups were collected to isolated total RNA, and performed to sequence on the Illumina Hiseq 3000 HTS platform (RiboBio Co. Ltd, Guangzhou, China). Differentially expressed transcripts were identified based on the criteria of fold change >1.5, and *p* < 0.05 and enriched via KEGG analysis.

### 4.8. Molecular Docking Study

Molecular docking was performed using the CDOCKER method embedded in Accelrys Discovery Studio 2.5 (AccelrysInc., San Diego, CA, USA) with default parameters. For TLR4 ligand preparation, Molecular Operating Environment (MOE2008) was used to convert the two-dimensional structures of candidates into three-dimensional structural data. The TLR4 protein structure was downloaded from the Protein Data Bank (accession code: 2Z64). In Accelrys Discovery Studio 2.5, hydrogen atoms were first added and all monosaccharide and water molecules were removed. The partial charges for each atom in TLR4 were assigned by CHARMm forcefield. The cavities in TLR4 were carefully searched for probable binding sites of inhibitors, and the biggest cavity, composed of Ile107, Asn129, Val131, Lys153 and His178 was selected as the binding site. The best docking poses of **1** or TAK-242 with the most negative docking scores were retained.

### 4.9. RT-PCR

RAW 264.7 macrophages were cultured in six-well plates at a density of 1.0 × 10^6^ cells/plate for 24 h. The cells treated with LPS (1 μg/mL) served as the model group, those treated with **1** and LPS (1 μg/mL) served as the treatment group, and those treated with 0.05% DMSO served as the control group (all for 24 h). Total RNA was isolated using the Trizol reagent (Invitrogen, Grand Island, NY, USA), and the concentration of RNA was measured using a Nanodrop 2000 ultramicro spectrophotometer (Thermo Fisher Scientific, Sacramento, CA, USA). The mRNA was reverse-transcribed into first-strand cDNA using the HiScript II Q RT SuperMix for qPCR (Vazyme, Nanjing, China). Quantitative RT-PCR assays were conducted using Hieff™ qPCR SYBR^®^ Green Master Mix (Yisheng, Shanghai, China) in a LightCycler 96 Real-Time PCR System (Roche, Basle, Switzerland). Gene-specific oligonucleotide primers were used for RT-PCR. Glyceraldehyde 3-phosphate dehydrogenase (GAPDH) served as an internal control, and primer sequences were listed in Table 2. The PCR mixtures were preincubated at 95 °C for 5 min, followed by 40 cycles of denaturation at 95 °C for 10 s, annealing at 55 °C for 20 s, and elongation at 72 °C for 20 s. All experiments were performed in triplicate.

### 4.10. Determination of Pro-Inflammatory Cytokine Levels

RAW 264.7 macrophages were seeded into 12-well plates at a density of 5 × 10^4^ cells/well for 24 h. After treatment with **1** (10 μM) or TAK-242 (10 μM) and stimulation with LPS (1 μg/mL) for 24 h, The release of IL-6 and TNF-α in cell supernatants was assayed using an ELISA kit (Boster, Wuhan, China), according to manufacturer’s instructions.

### 4.11. Western Blotting

RAW 264.7 macrophages were treated as same as RT-PCR. The cells were gently washed twice with ice-cold PBS, and lysed in Pierce^TM^ RIPA Buffer (Thermo Fisher, San Diego, CA, USA). Proteins were collected by centrifugation at 4 °C, 12,000× *g* for 10 min. Total proteins content was determined using a Pierce^TM^ BCA Protein Assay Kit (Thermo Fisher, San Diego, CA, USA). Subsequently, proteins were subjected to sodium dodecyl sulfate-polyacrylamide gel electrophoresis, and then transferred onto polyvinylidene fluoride membranes (Biosharp, Beijing, China). The membranes were blocked with 5% nonfat milk for 2 h at room temperature. The blocked membranes were then incubated with the primary antibodies recognizing TLR4 (Proteintech, Rosemont, IL, USA), MyD88 (Cell Signaling Technology, Danvers, MA, USA), p65, p-p65, IκBα, p-IκBα, iNOS and COX-2 (Abcam, Cambridge, UK), The reaction was allowed to proceed, at 4 °C for overnight, after which the membranes were incubated with secondary antibodies for 1.5 h at room temperature. The immunoreactive proteins on the blots were detected using an enhanced chemiluminescence detection system (Tanon, Shanghai, China). β-actin (Cell Signaling Technology, Danvers, MA, USA) was used as the internal control, and band intensities were quantified using Image-J.

### 4.12. Statistical Analysis

Values are presented as means ± standard deviation (SD) (n = 3). Statistical analyses were performed using one-way analysis variance (ANOVA) in SPSS 18.0. *p* < 0.05 indicated statistical significance.

## 5. Conclusions

**Figure 7:**
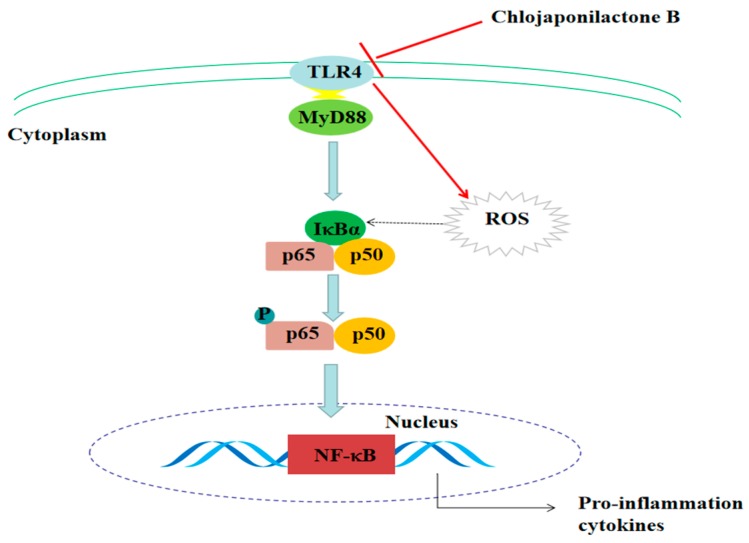
Scheme summarizing that **1** attenuated LPS-induced inflammatory responses by suppressing TLR4-mediated ROS generation and NF-κB signaling pathway.

To summarize, as shown in Figure 7, the present study revealed that **1** bound with the TLR4 protein, leading to the suppression of the inflammatory response, which decreased the production of ROS, inhibited the transcription of NF-κB, and eventually decreased the expression of inflammatory cytokines iNOS, NO, COX-2, IL-6 and TNF-α. It provided the evidence that **1** should be considered as a potential anti-inflammatory compound in future research.

## Figures and Tables

**Table 1:** Heat map showing various anti-inflammatory pathways.

Serial Number	Anti-Inflammatory Pathways
[1]	Toll-like receptor signaling pathway
[2]	MAPK signaling pathway
[3]	Inflammatory bowel disease (IBD)
[4]	NOD-like receptor signaling pathway
[5]	NF-κB signaling pathway
[6]	PPAR signaling pathway
[7]	AMPK signaling pathway
[8]	Jak-STAT signaling pathway
[9]	PI3K-Akt signaling pathway
[10]	Inflammatory mediator regulation of TRP channels
[11]	TNF signaling pathway
[12]	Rheumatoid arthritis

**Table 2:** Primer sequences of different inflammatory mediators.

Gene Symbol	Forward Primer (5′-3′)	Reverse Primer (5′-3′)
GADPH	GGTGAAGGTCGGTGTGAACG	CTCGCTCCTGGAAGATGGTG
TLR4	AAATGCACTGAGCTTTAGTGGT	TGGCACTCATAATGATGGCAC
MyD88	ATCGCTGTTCTTGAACCCTCG	CTCACGGTCTAACAAGGCCAG
iNOS	GGAGTGACGGCAAACATGACT	TCGATGCACAACTGGGTGAAC
COX-2	ATCCCAACAAACGACCTAAA	CAGAACGACTCGGTTATCAA
IL-6	AGTCACAGAAGGAGTGGCTAA	GGCATAACGCACTAGGTTT
TNF-α	ACAGCCAGGCTTCGTTTAGG	GCCAATTTCGGACTCAGCATC
NF-κB	ATGGCAGACGATGATCCCTAC	TGTTGACAGTGGTATTTCTGGTG

## Supplementary Materials

NMR (^1^H, ^13^C) spectra of compound **1**, and the method and results of cytotoxicity test on compound **1** and TAK-242.
