# Supplementary material for: Chlojaponilactone B Attenuates Lipopolysaccharide-Induced Inflammatory Responses by Suppressing TLR4-Mediated ROS Generation and NF-κB Signaling Pathway
Source: Molecules. 2019 Oct 16;24(20):3731. doi: 10.3390/molecules24203731 (PMC6832138; doi:10.3390/molecules24203731)
Supplement: Supplementary file 1 [file molecules-24-03731-s001.pdf]

Chlojaponilactone B Attenuates Lipopolysaccharide-Induced Inflammatory Responses by Suppressing TLR4-Mediated ROS Generation and NF-κB Signaling Pathway

Shaoxia Ye [1], Qiyao Zheng [1], Yang Zhou [1], Bai Bai [1], Depo Yang [1,2] and Zhimin Zhao [1,2,*]

[1] School of Pharmaceutical Sciences, Sun Yat-sen University, Guangzhou, Guangdong 510006, People's Republic of China

yeshx9@mail2.sysu.edu.cn (S.Y.); zhengqy5@mail2.sysu.edu.cn (Q.Z.); zhouy596@mail2.sysu.edu.cn (Z.Y.); baib6@mail2.sysu.edu.cn (B.B.); lssydp@mail.sysu.edu.cn (D.Y.); zhaozhm2@mail.sysu.edu.cn (Z.Z.)

[2] Guangdong Technology Research Center for Advanced Chinese Medicine, Guangzhou, Guangdong 510006, People's Republic of China

* Correspondence: zhaozhm2@mail.sysu.edu.cn; Tel.: +86-020-39943043

Figure	Content	Page
S1	^{1}H NMR spectrum of chlojaponilactone B (**1**)	3
S2	^{13}C NMR spectrum of chlojaponilactone B (**1**)	4
S3	Cytotoxicity Test	5
S4	Figure S1. Determination of the cytotoxic effects of 1 and TAK-242	6

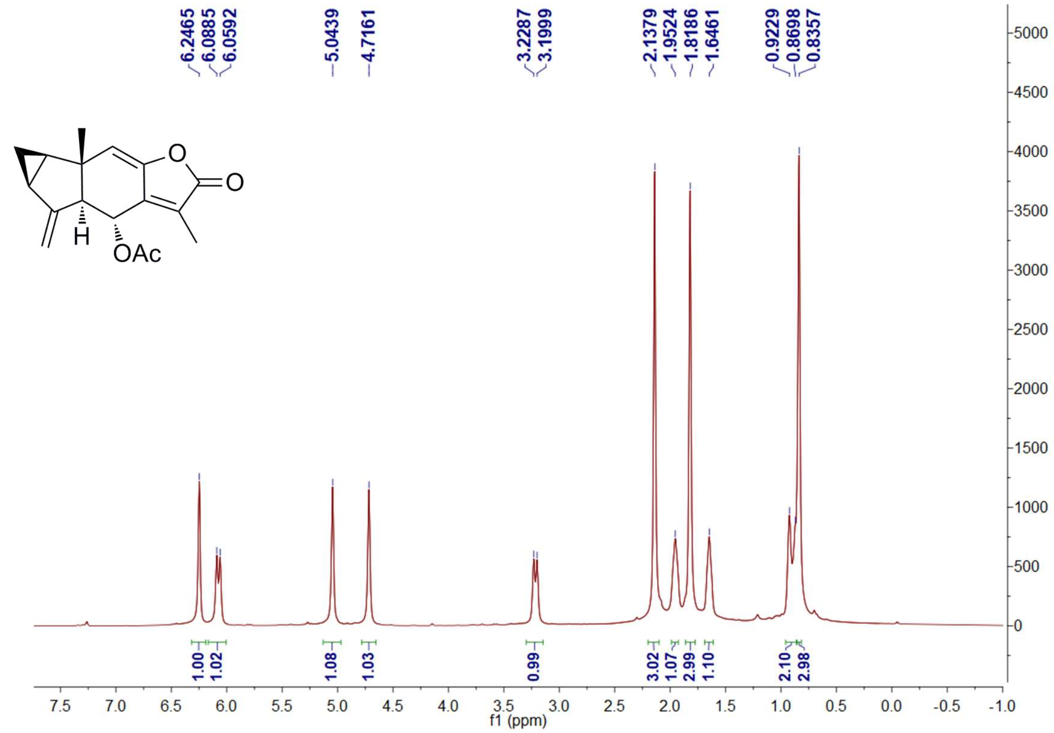

S1. ^{1}H NMR spectrum of chlojaponilactone B (**1**) ($CDCl_3$, 400 MHz)

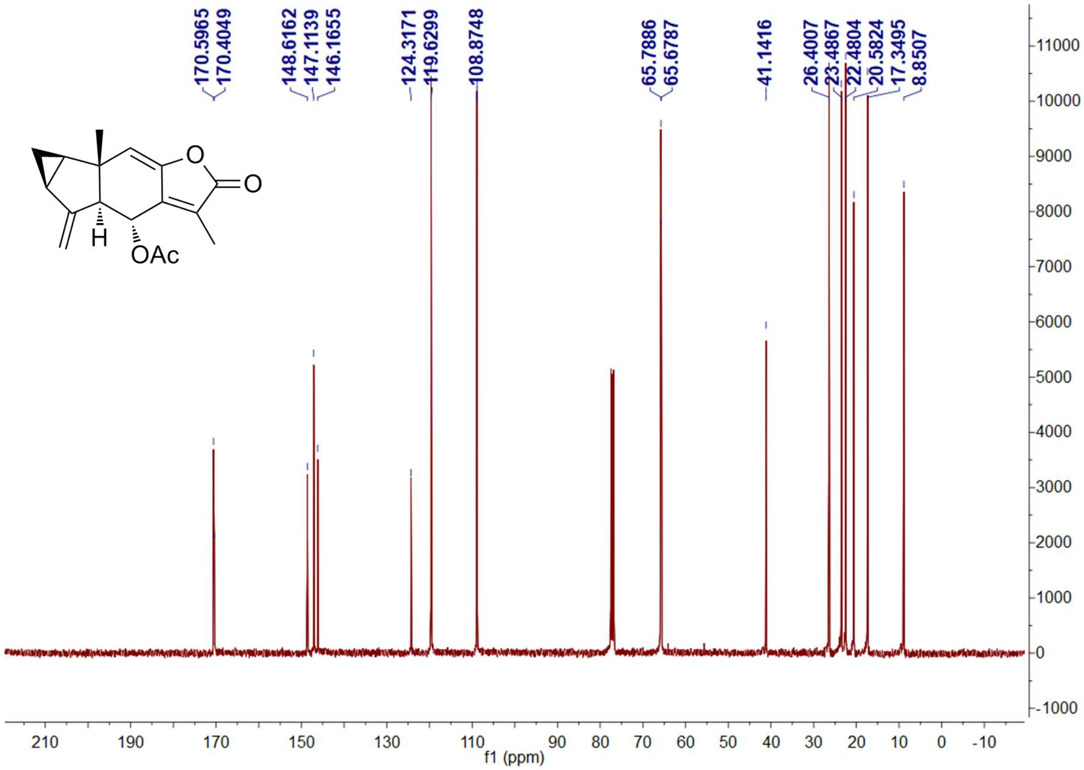

S2. ^{13}C NMR spectrum of chlojaponilactone B (**1**) ($CDCl_3$, 100 MHz)

S3. *Cytotoxicity Test*

Cell viability was analyzed using the 3-(4,5-dimetrylthiazol)-2,5-diphenyltetrazolium bromide (MTT) assay. RAW 264.7 macrophages were seeded at a density of 5×10^3 cells/well in 96-well plates for 24 h. The cells were then treated with **1** or TAK-242 at various concentrations with or without LPS (1 μg/ml) for 24h. An equal concentration of a solvent vehicle (DMSO, 0.5%) was included as the control. MTT (5 mg/mL in sterile PBS) solution was added to the culture (20 μL/well), followed by incubation for 4 h. Finally, the medium was removed, 100 μL DMSO was added to each well, and absorbance was measured at 490 nm by using a microplate reader (Molecular Devices, Sunnyvale, CA, USA).

S4.

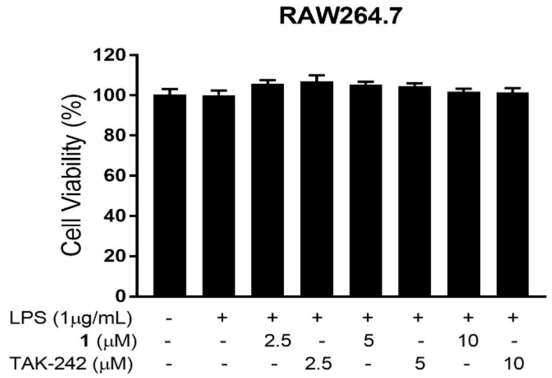

Figure S1. Determination of the cytotoxic effects of **1** and TAK-242 in LPS-induced RAW264.7 cells. Cells were incubated with increasing concentrations of **1** (2.5, 5 or 10 μM) or TAK-242 (2.5, 5 or 10 μM) and treated with LPS (1 μg/mL). The cell viability was examined with the MTT assay.
